# Simultaneous measurement of methane, propane and isobutane using a compact mid-infrared photoacoustic spectrophone

**DOI:** 10.1016/j.pacs.2024.100635

**Published:** 2024-07-30

**Authors:** Huaiyu Mei, Gaoxuan Wang, Yinghe Xu, Haijie He, Jun Yao, Sailing He

**Affiliations:** a Centre for Optical and Electromagnetic Research, Ningbo Innovation Center, College of Optical Science and Engineering, Zhejiang University, Hangzhou 310058, China; b Taizhou Hospital, Zhejiang University, Taizhou, China; c National Engineering Research Center for Optical Instruments, Zhejiang University, Hangzhou 310058, China; d Taizhou University, Taizhou, 318000, China; e Department of Electromagnetic Engineering, School of Electrical Engineering, Royal Institute of Technology, 10044 Stockholm, Sweden

**Keywords:** Photoacoustic spectroscopy, Multi-gas sensing, Wavelength-modulation, Second harmonic signal, Propane, Methane

## Abstract

Hydrocarbon gas sensing is a challenging task using laser absorption spectroscopy due to the complex and broad structure of absorption lines. This application requires quick, accurate and highly sensitive detection of hydrocarbon gases concentrations. In this paper, a compact photoacoustic spectrophone was developed to simultaneously measure methane, propane and isobutane. This spectrophone uses wavelength modulation spectroscopy (WMS) with a single acoustic resonator and a single DFB laser emitting at 3368 nm, which greatly reduces the system complexity without using time-division multiplexing technology for multi-gas sensing. Due to the complex and broadband absorption of hydrocarbon gases, a novel signal processing method based on multilinear regression with Ridge regression (MLR-RG) is proposed to reduce the measurement error caused by the nonlinearity of spectra signal. For single gas measurement, the detection limits of methane, propane, and isobutane are determined to be 828 ppb, 419 ppb, and 619 ppb (SNR = 1, integration time = 20 s), respectively. For simultaneous multi-gas sensing in a gaseous mixture, the detection limits of propane and isobutane are determined to be 7 ppb, 68 ppb with an integration time of 860 s, 460 s, respectively. The measurement accuracy of propane and isobutane using MLR-RG is higher than that of ordinary least squares regression and partial least squares regression by 75% and 60%, respectively. The proposed algorithm based on MLR-RG provides a promising approach to process the broad overlapping absorption spectra for accurately retrieving hydrocarbon gases concentrations.

## 1 Introduction

Hydrocarbon gases including methane (CH_4_), ethane (C_2_H_6_), propane (C_3_H_8_), propene (C_3_H_6_), isobutane (C_4_H_10_) et al. are highly flammable and explosive, and play crucial roles in the field of petrochemical industry, chemical manufacturing industry and pharmaceuticals production. They are commonly used as fuel, refrigerant and organic solvent [1], [2], [3]. Many safety accidents occurred in coal mines due to methane explosions [4]. Propane is a common industrial fuel and used as an anesthetic in surgery [5]. Isobutane as important fuel is a common refrigerant that has significant advantages over traditional Freon-based refrigerants [6]. Therefore, it is of great significance to monitor the concentration of hydrocarbon gases such as methane, propane and isobutane in the petrochemical industry and chemical manufacturing industry in order to avoid accidents.

Optical absorption spectroscopy has proven to be a perfect candidate in trace gas sensing, which are mainly classified as tunable diode laser absorption spectroscopy (TDLAS) [7], [8], [9], non-dispersive infrared spectroscopy (NDIR) [10], cavity enhanced absorption spectroscopy (CEAS) [11], photoacoustic spectroscopy (PAS) [12], [13], [14], [15], [16]. TDLAS technique usually requires a long optic-path cavity such as White or Herriott cells to improve spectral signal, leading to a complex and bulky device [17]. NDIR with advantages of smaller size and lower cost presents poor stability due to broadband light sources [18], [19]. CEAS technology requires PDH (Pound–Drever–Hall) or optical feedback interference cancellation techniques which adds complexity to the system [20], [21].

PAS has attracted more attention due to its advantages of high sensitivity, fast response, good stability, and small gas absorption cell volume [22]. PAS has been widely used in environmental monitoring [23], [24], [25], [26], [27], [28], medical diagnosis (breath analysis) [29], [30], [31], and industrial production [32], [33]. Two commonly used transducers in PAS are quartz tuning fork (QTF) and microphone. QTF-based photoacoustic spectroscopy is referred to as quartz-enhanced photoacoustic spectroscopy (QEPAS) with advantages of small size, low cost and high Q factor, but also with the risk of corroding the silver layer on QTF to degrade the measurement performance [34], [35], [36], [37]. Light-induced thermoelectric spectroscopy (LITES) is a novel QTF-based gas sensing technology that utilizes light-thermal elastic conversion on QTF to retrieve gas concentration. The QTF in LITES technology is not directly exposed with the target gas, which realizes non-contact measurement [38], [39]. Microphone-based PAS method is commonly associated an acoustic resonance cavity for amplifying acoustic signal, which is a stable and mature solution for high-sensitive sensing of trace gas [22], [23], [24], [25], [26], [27], [28], [29], [30], [31].

For multi-gas sensing, many strategies based on PAS have been reported recently, which are roughly classified into two categories: multi-resonator or multi-laser, or adopting a broadband light source for covering several absorption lines. Besson et al. used multiple lasers in 1651 nm, 1742 nm 1369 nm to sequentially scan in a single resonator to measure CH_4_, HCl, and H_2_O [40]. Hanyecz et al. used dual lasers and dual resonators to measure CH_4_, H_2_O, CO, and CO_2_ [41]. Wang et al. used several tunable laser sources combined with time-division multiplexing technology to measure H_2_O, C_2_H_2_, CO, and CO_2_ [42]. The approach of multi-acoustic resonator or multi-laser require a complex gas sensing system and increase the cost due to several sets of laser controller. A single light source for multi-gas sensing must be broadband for covering multiple absorption lines, which might deteriorate measurement accuracy due to the overlapping spectra from different trace gases [33]. Advanced signal processing methods, are recently reported for improving the measurement accuracy and eliminating the measurement interference from multiple gases, which mainly includes optimized time-division multiplexing [43], signal aliasing processing [44], multiple linear regression [45] and partial least squares regression [46]. For the spectral signal with distinguishable absorption lines and satisfying linear superposition, least squares regression and partial least squares regression can effectively recognize the intensity of each absorption peak to retrieve the trace gas concentration [47]. However, the spectral signal generated by trace gases with broadband absorption spectrum exhibit severe aliasing, which is not completely linearly correlated with the concentration of individual gases. Linear regression algorithms directly dealing with such broadband spectral signal can lead to significant deviations. To avoid reducing measurement accuracy due to signal nonlinearity, multiple linear fitting based on ridge regression are used to retrieve the gas concentrations. Ridge regression is more stable and applicable than ordinary least squares regression [48]. A weight matrix in ridge regression is introduced to focus on the linear featured spectral signal point and reduce the impact from the nonlinear signal points. The proposed method of multiple linear fitting based on ridge regression is experimentally validated by measuring the broad-spectrum absorbing gases of methane, propane and isobutane.

In this paper, a compact synchronous photoacoustic spectrometer for measurement of methane, propane, and isobutane with broadband absorption spectrum is presented. This spectrometer is based on WMS with a single acoustic resonator and a single DFB laser emitting at 3368 nm, which greatly reduces the system complexity without using time-division multiplexing technology for multi-gas sensing within a single scan cycle (20 s). Due to the broadband absorption of propane and isobutane, a new signal processing method called multilinear regression based on Ridge regression (MLR-RG) for separating the absorption signals of individual trace gas was proposed. For single gas measurement, the detection limits of CH_4_, C_3_H_8_, and C_4_H_10_ are determined to be 828 ppb, 419 ppb, and 619 ppb (SNR = 1, integration time = 20 s), respectively. For simultaneous multi-gas sensing in a gaseous mixture, the limit of detection of propane and isobutane are determined to be 7 ppb (integration time = 860 s), 68 ppb (integration time = 460 s), respectively. The measurement accuracy of propane (10 ppm) and isobutane(10 ppm) using MLR-RG is higher than that of ordinary least squares regression and partial least squares regression by 75%, and 60%, respectively.

## 2 Theory

### 2.1 Photoacoustic spectroscopy

In PAS, a periodic thermal wave is generated due to the sample absorption of power-modulated laser light. This modulated thermal wave transfers to the surrounding and usually resonates with an acoustic cavity mode to form a standing acoustic wave. The amplitude of the standing acoustic wave is proportional to the absorber concentration. The acoustic signal in the resonance cavity can be expressed as [22].

(1) S=C⋅P(λ)α(λ)

where C is the cell constant, which is related to the resonance cavity shape, acoustic mode, laser beam profile, and microphone response. P is laser power, α=(Nσ) is the absorption coefficient, which is related to the number density of molecules N and absorption cross-section σ. In wavelength modulation PAS, the acoustic signals are generated in the cell due to the absorption of modulated light. The acoustic eigenmodes of the closed cavity are the solutions of the homogeneous wave equation and are expressed as [49].

(2) $$A_{n}=\frac{\left(\zeta -1\right)LF_{n}Q_{n}}{V_{res}\omega_{n}}\alpha P$$

ζ represents the gas adiabatic coefficient, L is the length of the resonance cavity, $F_{n}$ is the dimensionless normalized overlap integral value, $Q_{n}$ is the quality factor of the nth harmonic, $V_{res}$ is the volume of the resonance cavity, and $\omega_{n}$ is the nth harmonic resonant angular frequency. The acoustic resonance cavity acts as an acoustic amplifier to amplify the modulated acoustic signal. The excited acoustic signal is captured by a microphone and measured by a lock-in amplifier to retrieve the gas concentration.

### 2.2 Multilinear Regression Based on Ridge Regression (MLR-RG)

Least squares regression and ridge regression are both linear regression, which assume that the independent variable and dependent variable are linearly related and satisfy the linear regression equation as following.

(3) $$y=\omega_{0}+\omega_{1}x_{1}+\cdots +\omega_{n}x_{n}$$

The purpose of linear regression is to find a matrix $W=\left[\omega_{0},\omega_{1},\ldots ,\omega_{n}\right]$ such that WX approximates Y. The ordinary least squares regression assumes that the data is unbiased, which results in an optimal solution that minimizes the objective function as shown:

(4) $$S^{2}={\left(WX-Y\right)}^{2}={\left(Y-WX\right)}^{\prime }\left(Y-WX\right)=Y^{\prime }Y-2W^{\prime }X^{\prime }Y+W^{\prime }X^{\prime }WX\to min$$

To derive the minimum value of $S^{2}$, we take a derivative of $S^{2}$ with respect to W as shown:

(5) $$\frac{\partial S^{2}}{\partial W}=0\to X^{\prime }XW=X^{\prime }Y\to W={\left(X^{\prime }X\right)}^{-1}X^{\prime }Y$$

However, due to the issue of multicollinearity, the matrix (W) is not full rank, which leads to significant computational errors without a unique solution, and a certain deviation between the observed data and the real data due to noise. The ordinary least squares regression lacks in dealing with abnormal data. Ridge regression, as an improved model of ordinary least squares method, presents an addition of a penalty term ($\alpha W^{\prime }W$) to the objective function as following [50]:

(6) $$S^{2}={\left(WX-Y\right)}^{2}+\alpha W^{\prime }W=Y^{\prime }Y-2W^{\prime }X^{\prime }Y+W^{\prime }X^{\prime }WX+\alpha W^{\prime }W$$

(7) $$\frac{\partial S^{2}}{\partial W}=0\to W={\left(X^{\prime }X+\alpha E\right)}^{-1}X^{\prime }Y$$

Where E is an identity matrix. α is a positive parameter. A greater α presents a more robust fit. Ridge regression can yield a model with a robust W, while least squares regression may produce multiple different models in dealing with the problem of multicollinearity. Ridge regression can not only alleviate the problem of multicollinearity (in which case $X^{\prime }X+\alpha E$ is inverted to solve Eq. (7)), but also enhance the fitting capability for abnormal data. Because of the α restrictions on matrix W in the objective function (also known as L2 constraints or L2 regularization in the field of machine learning), the Ridge regression algorithm tries to minimize the magnitude of W, which makes Ridge regression more stable and applicable compared to ordinary least squares regression [48].

MLR-RG is proposed to fitting the featured spectral signal points. A weight matrix (M) is used to highlight the linear featured spectral signal points. This approach further reduces the nonlinear errors in the fitting of spectral data. the weight matrix is determined as follows:

(8) $$M=1-\beta \times \frac{S-S_{1}-S_{2}-\cdots -S_{n}}{S}$$

Where S represents the mixture spectral signal, and $S_{1},S_{2},\ldots ,S_{n}$ are the spectral signals of individual gas. β is a magnification that focuses on the adjustment of the weight matrix. The larger β corresponds to a smaller weight (M) for the same error. For signals that fully satisfy linear superposition, the weight matrix M would be a matrix of all ones. For signals exhibiting nonlinear distortion, the values in the M matrix range from 0 to 1. The values in the M matrix is higher in regions where the linear superposition is better. By using the weight matrix, the nonlinear errors are reduced in the signal superposition. this method is used to address the issue of nonlinear superposition in the photoacoustic signal of trace gases (methane, propane and isobutane) with broad absorption spectra.

**Fig. 1:**
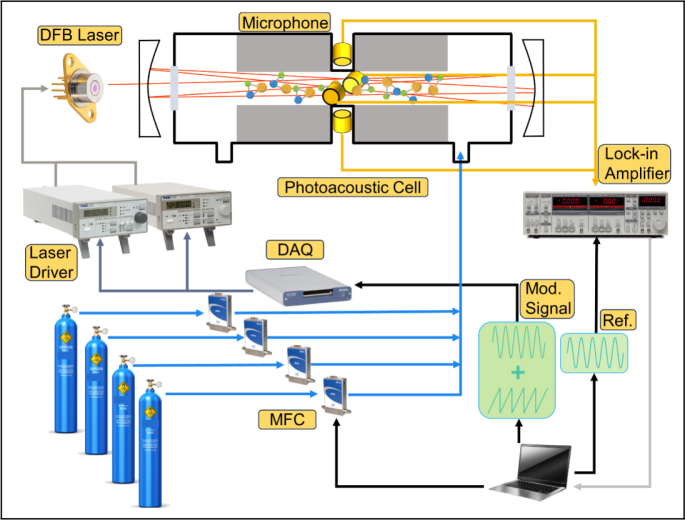
Structural diagram of experimental device for gas mixing measurement; the laser driver includes a current and temperature controller for driving the DFB laser; the photoacoustic cell consists of two buffer zones and a cylindrical resonator, four microphones are placed to detect the photoacoustic signals; the lock-in amplifier is used to extract harmonic signals, and the modulation signal is generated by a data acquisition card controlled by a PC.

## 3 Experiment setup

Fig. 1 shows the schematic diagram of the developed wavelength modulation photoacoustic spectrometer using a single DFB laser (Nanoplus) with a central wavelength of 3368 nm. The DFB laser is precisely controlled using a temperature controller (TED200C, Thorlabs) and a current controller (LDC 205C, Thorlabs). The temperature of DFB laser is set at 13 ℃. the injected current is composed of a low-frequency sawtooth wave superimposed by a high-frequency sine wave. The modulated current signal is generated using a data acquisition card. The sawtooth wave signal covers the absorption spectra of methane, propane, and isobutane. The high-frequency sine signal modulates the laser wavelength, which leads to a periodic change in the absorption intensity of gases to the laser and generates a periodical acoustic pressure signal with an identical frequency. The gas is controlled by a mass flow meter (GE50 A, MKS) and enters the photoacoustic cell and the mass flow meter is controlled by a computer. The photoacoustic cell is made of stainless steel 304 without coating, consists of two buffer zone and a cylindrical resonator with a length of 23 mm and 8 mm in diameter, the buffer zone can effectively reduce airflow noise.

**Fig. 2:**
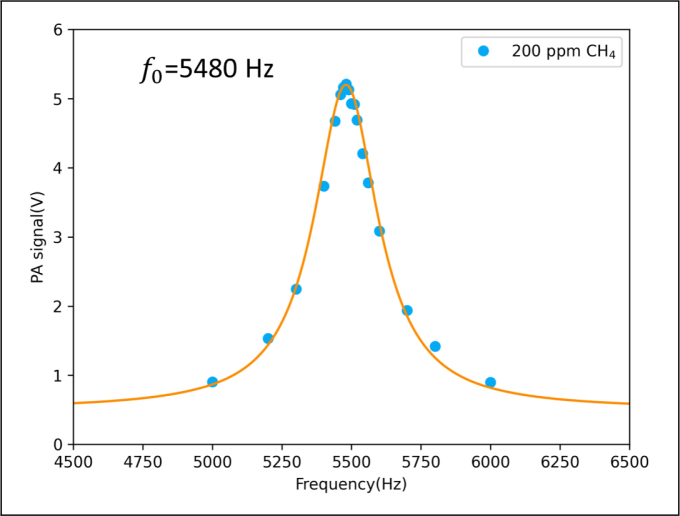
Frequency response curves of PA cell; using 200 ppm methane gas for measurement, different modulation frequencies were set between 2500–3000 Hz. To extract the second harmonic signal, the demodulation frequency of the lock-in amplifier was set to 5000–6000 Hz; optimal resonance frequency is 5480 Hz.

Fig. 2 shows the frequency response of the photoacoustic cell, where wavelength modulation technique was used to measure methane with concentration of 200 ppm. Lorentzian fit was applied and the full width half maximum (FWHM) is 280 Hz leads to a quality factor Q of 19.6 (=5480/280), and the optimal resonant frequency of the photoacoustic cell was determined to be 5480 Hz.

There are two reflecting mirrors placed outside the photoacoustic cell, which enhance the photoacoustic signal through three reflections. Four microphones are placed in the middle of the photoacoustic cell to extract photoacoustic signals. The microphone signals are connected to a lock-in amplifier (SR830, SRS) to extract the second harmonic signal. The laser is directly driven by the temperature and current controller (TED200C and LDC 205C, Thorlabs). The laser temperature is set at 13 ℃. A data acquisition card (USB6361, NI) is used to generate a sawtooth signal for scanning the laser frequency and a high-frequency sine wave for modulating the laser power. The output signal of the lock-in amplifier is collected by the data acquisition card and displayed on a computer in real time.

**Fig. 3:**
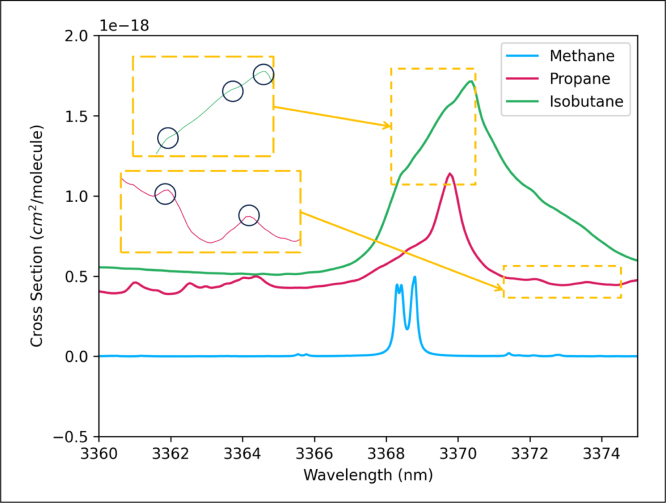
Cross section data of three gases at the conditions of 298.1 K and 760.0 Torr; the data was obtained from the HITRAN database using an API interface for loading data; the absorption cross-section was calculated using the Lorentz line shape function.

According to the high-resolution transmission molecular absorption database (HITRAN) [51], the absorption cross-sections of three gases are plotted as shown in Fig. 3. The laser wavelength scanning range is set to 3367–3371 nm to cover the absorption spectra of the three gases.

The absorption cross section in Fig. 3 is not a smooth curve, but shows many bumps or depressions. These bumps and depressions can cause suddenly increase or decrease in the photoacoustic signal, which results in multiple second harmonic signals that are used to reconstructed the material concentration.

## 4 Results and discussion

### 4.1 Optimization of modulation amplitude and PA signal linearity

**Fig. 4:**
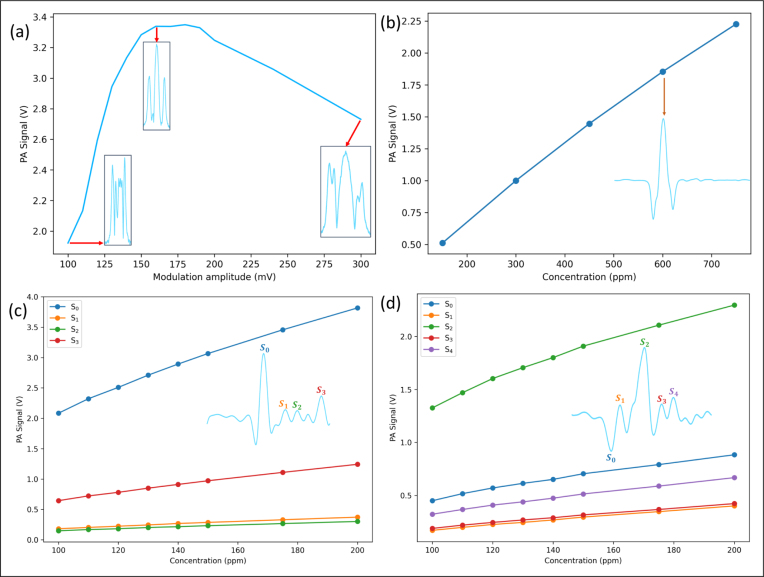
(a) Modulation amplitude and waveform, photoacoustic signal relationship diagram; using 1500 ppm methane, only the modulation amplitude was varied while keeping all other conditions constant. (b) methane concentration and photoacoustic signal relationship diagram; the flow rate ratio between nitrogen and methane gases was adjusted to vary the concentration of methane in PA cell. (c) propane concentration and photoacoustic signal relationship diagram. (d) isobutane concentration and photoacoustic signal relationship diagram.

Methane with a concentration of 1500 ppm is used for optimizing the modulation amplitude. The laser wavelength scanned two absorption peaks of methane as shown in Fig. 3. The secondary harmonic waveforms amplitude versus the modulation amplitude are shown as Fig. 4(a). The photoacoustic signal shows multiple peaks due to the overlap of the two absorption lines with the modulation amplitude of less than 100 mV. The photoacoustic signal wave is a typical secondary harmonic signal in the modulation amplitude range of 150–200 mV, as two absorption peaks with close position are approximated as one absorption line with high modulation amplitude. The maximum PA signal tends to be saturated in the range of 150–200 mV. When the modulation amplitude increases to more than 200 mV, the signal spectrum becomes wider and the peak value becomes smaller. According to the curve data, the optimal modulation amplitude is determined to be 160 mV, which leads to a maximum secondary harmonic amplitude and avoids signal distortion. The linearity test of absorption signals of three gases with modulation amplitude of 160 mV is carried out as shown in Fig. 4(b–d). The inset map in Fig. 4(c) shows a typical photoacoustic signal for measuring 100 ppm propane. Four peaks points ($S_{0}$, $S_{1}$, $S_{2}$, $S_{3}$ in Fig. 4(c)) are used to plot the relationship between PA signal amplitude and the concentration. For isobutane, the illustration in Fig. 4(d) presents the photoacoustic signal of 100 ppm. Five peaks values ($S_{0}$, $S_{1}$, $S_{2}$, $S_{3}$, $S_{4}$ in Fig. 4(d)) are used to demonstrate the linear relationship of PA signal versus isobutane concentration. Table 1 summaries the linearity of photoacoustic signal vs. different concentrations with a regression coefficient > 0.98. It should be noted that propane and isobutane have broadband absorption spectra (as shown in Fig. 3) and consequently their 2f PA signals have multiple peak points (due to the variation of their absorption cross-section in the broadband absorption spectra), which are different from the typical 2f signal generated by a narrow-band absorbing gas.

After measuring the linearity of methane, propane, and isobutane, high-purity nitrogen was continuously introduced into the photoacoustic cell for five minutes before measuring the noise signal. By calculating the standard deviation of the noise signal, the noise 1σ of methane, propane, and isobutane were determined to be 2.659 mV, 8.734 mV, and 8.204 mV (the sensitivity of lock-in amplifier for methane is set to 1000 mV, while for propane and isobutane, it is set to 200 mV). For single gas measurement, using the data from Table 1, the detection limits (SNR = 1, integration time = 20 s) for methane, propane, and isobutane were determined to be 828 ppb, 419 ppb, 619 ppb.

**Table 1:** Linear fit of PA signal and concentration.

Sensing_region	PA signal (mV) vs. concentration (ppm) C	R2
CH_4_	S=21.4⋅C+122.5	0.9972
C_3_H_8__$S_{0}$	S=17.2⋅C+441.7	0.9935
C_3_H_8__$S_{1}$	S=1.92⋅C−5.992	0.9966
C_3_H_8__$S_{2}$	S=1.53⋅C−0.05515	0.9967
C_3_H_8__$S_{3}$	S=5.94⋅C+68.93	0.9968
C_4_H_10__$S_{0}$	S=4.22⋅C+53.59	0.9896
C_4_H_10__$S_{1}$	S=2.28⋅C−51.96	0.9977
C_4_H_10__$S_{2}$	S=9.53⋅C+437.1	0.9854
C_4_H_10__$S_{3}$	S=2.30⋅C−33.70	0.9968
C_4_H_10__$S_{4}$	S=3.40⋅C−5.567	0.9963

### 4.2 MLR based on ridge regression for simultaneous measurement of propane and isobutane

**Fig. 5:**
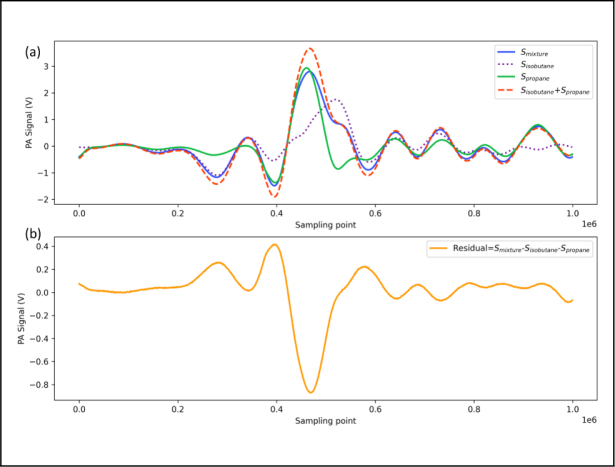
(a) The photoacoustic signals of the two gases individually and the photoacoustic signal of the mixture, where $S_{propane}+S_{isobutane}$ represents the superimposed value of the photoacoustic signals of the two gases, which differs from the photoacoustic signal of the mixture, $S_{mixture}$ is the second harmonic of the mixture. (b) The residual between the photoacoustic signal of the mixture and the individual photoacoustic signals of the two gases, which is non-zero, indicates that the photoacoustic signals do not exhibit a linear superposition relationship.

Fig. 5(a) shows the recorded photoacoustic signal caused by the light absorption of 150 ppm propane (green line), 150 ppm isobutane (short dash line) and the two gas mixtures (blue line), respectively. The long dashed red line in Fig. 5(a) presents the linear summation of PA signal caused by 150 ppm propane and 150 ppm isobutane, which shows differences compared with PA signal of the mixture (the blue line). Fig. 5(b) plots the residual between the blue line and the long dashed line, which indicates that the signal process using the spectral signal of mixtures without considering the complex absorption spectral feature would lead to an incorrect estimate of propane and isobutane concentration.

For accurate estimation of propane and isobutane concentration, MLR-RG was used for considering the significant overlapping of absorption spectral from propane and isobutane. The signal peaks shown Table 1 are used to retrieve the concentration of propane and isobutane.

Based on Fig. 5, the spectral signal sampling point with small residuals represents high linear correlation, which are the sampling points of $0.27\times 10^{6}$, $0.34\times 10^{6}$, $0.39\times 10^{6}$, $0.46\times 10^{6}$, $0.53\times 10^{6}$, $0.58\times 10^{6}$, $0.64\times 10^{6}$, $0.73\times 10^{6}$, $0.78\times 10^{6}$, $0.86\times 10^{6}$, $0.93\times 10^{6}$. Therefore, the above sampling points are used in the MLR-RG algorithm. Subsequently, the regression weight matrix will be computed. The regression weight (M) for each position is calculated as follows:

(9) $$M=1-\beta \times \frac{\left|S_{mixture}-S_{C_{3}H_{8}}-S_{C_{4}H_{10}}\right|}{S_{mixture}}$$

where $S_{mixture}$ refers to the photoacoustic signal of the mixed gas, $S_{C_{3}H_{8}}$ refers to the photoacoustic signal of propane, and $S_{C_{4}H_{10}}$ refers to the photoacoustic signal of isobutane.

The photoacoustic signals generated by the two types of broadband absorption gases mutually influenced each other, resulting in underestimated regression values. To eliminate this mutual interference between the two gases, we corrected the photoacoustic signals in the aforementioned fitted regression regions. For the photoacoustic signal for each individual gas, assuming there exists a linear relationship as follows.

(10) $$S_{mixture}=S_{C_{3}H_{8}}+S_{C_{4}H_{10}}-{ɛ}_{1}\times C_{C_{3}H_{8}}-{ɛ}_{2}\times C_{C_{4}H_{10}}$$

${ɛ}_{1}$ and ${ɛ}_{2}$ are determined by measuring the PA signals ($S_{C_{3}H_{8}}$ and $S_{C_{4}H_{10}}$) with propane concentration of 100 ppm and isobutane concentration of 150 ppm. PA signal of $S_{mixture}$ is excited by the mixture of 100 ppm propane and 150 ppm isobutane. The results are shown in Table 2, where the units of ${ɛ}_{1}$ and ${ɛ}_{2}$ are mV/ppm. At the sampling point of 462000, the two gases mutually weaken each other’s signals. At the sampling point of 530000, an increase in propane concentration reduces the photoacoustic signal at this location (the photoacoustic signal here is negative), while an increase in isobutane concentration enhances the photoacoustic signal at this point.

**Fig. 6:**
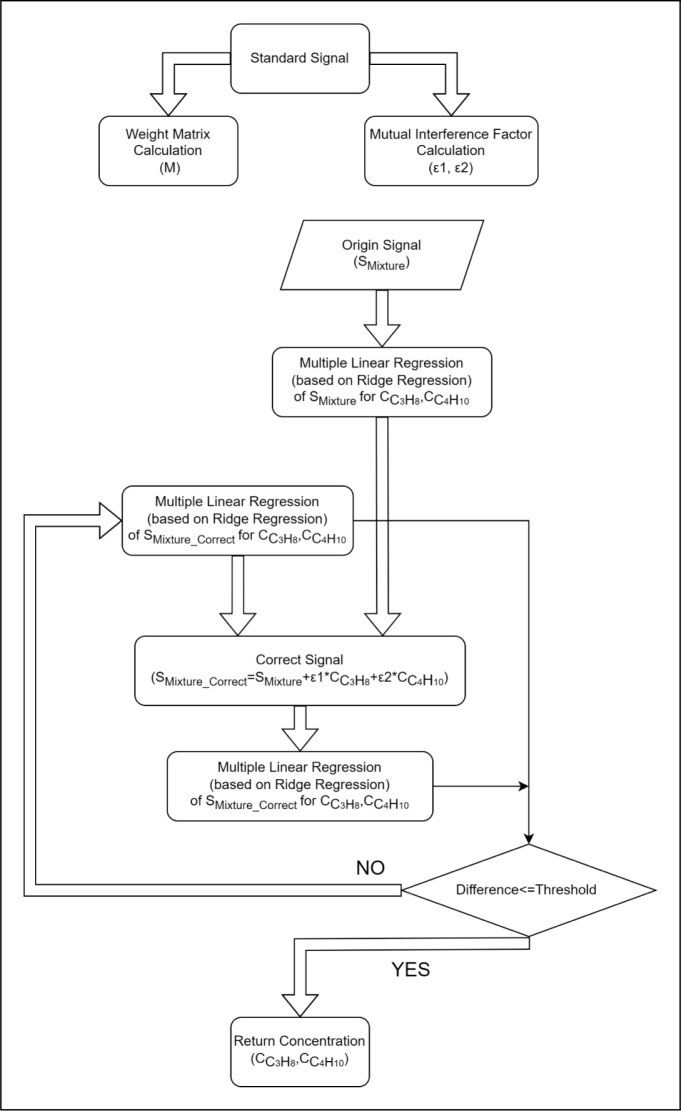
Flowchart of the MLR-RG, at the beginning of the calculation, use standard data to compute the weight matrix and interference factor, the results will be used in subsequent signal correction and data fitting. The original signal will first fit by MLR-RG, the outcome is similar to least squares regression. Subsequently, signal correction will be performed to compensate for the nonlinearities arising from the superposition of signals. then another fitting will be carried out, and the difference between the previous fitting result and the corrected fitting result will be compared. When the difference is less than a threshold value, the output will be generated.

Fig. 6 shows the flowchart of the program operation using MLR-RG. A weight matrix and two mutual interference factors are determined by the measured PA signals of propane 100 ppm and isobutane 150 ppm. By incorporating the weight matrix into the ridge regression, we obtained the initial concentrations of propane and isobutane ($C_{C_{3}H_{8}}$ and $C_{C_{4}H_{10}}$) with the observed original PA signal ($S_{mixture}$). With the initial concentrations of propane and isobutane ($C_{C_{3}H_{8}}$ and $C_{C_{4}H_{10}}$) and two mutual interference factors (${ɛ}_{1}$ and ${ɛ}_{2}$), a correction of original PA signal is achieved as shown in Fig. 6 for accurately retrieving the concentrations of propane and isobutane. A difference between the initial concentrations and the concentrations derived from the corrected PA signal ($S_{mixture\text{\_}correct}$) is obtained. The program is ended with the difference less than a set threshold, otherwise a new loop is reentered to achieve more accurate concentrations.

**Table 2:** Linear fit of PA signal and concentration.

Sampling point	${ɛ}_{1}$	${ɛ}_{2}$
277000	−0.08566	−1.553
340000	−0.5953	0.4022
396000	−0.2543	−2.397
462000	1.270	3.837
530000	−0.6906	0.8540
586000	−0.3483	−0.9854
639000	−0.1577	0.4794
732000	−0.1693	0.6673
785000	−0.09080	−0.3740
860000	0.04049	−0.5516
930000	−1.571	1.396

**Fig. 7:**
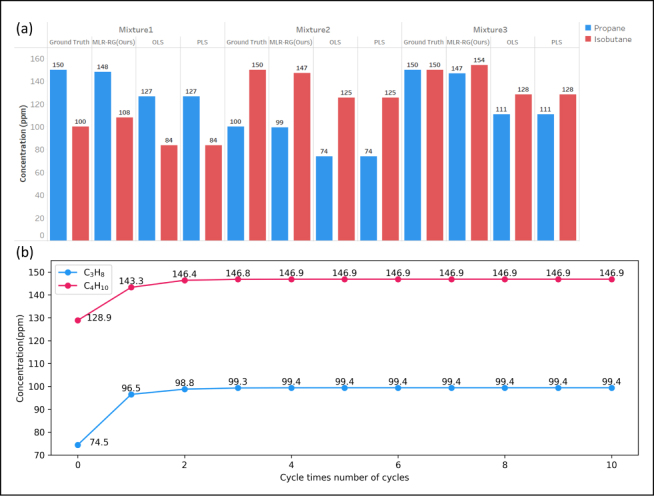
(a) Comparison diagram of detection results of propane and isobutane dual gases. (b) Changes in the results during the calculation of the algorithm; the result of the first fitting corresponds to the cycle time of 0. With each iteration, the degree of change in concentration decreases by an order of magnitude, ultimately reaching a convergent state.

**Fig. 8:**
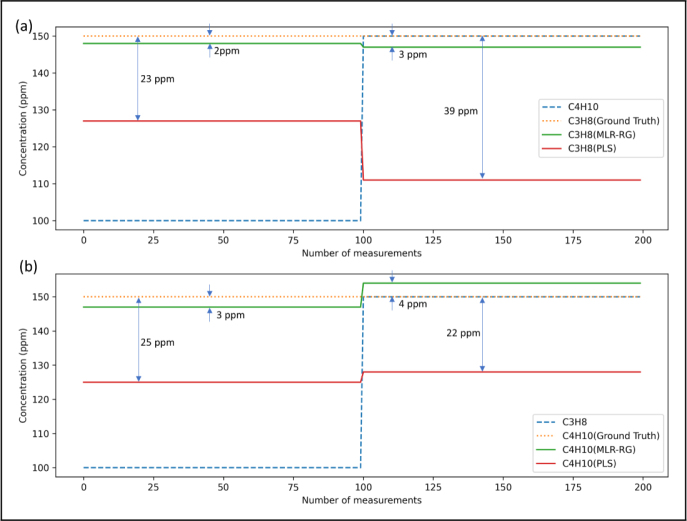
(a) Comparison of retrieving gas concentration using MLR-RG and PLS with constant propane concentration of 150 ppm and isobutane concentration from 100 ppm to 150 ppm. (b) Comparison of retrieving gas concentration using MLR-RG and PLS with constant isobutane concentration of 150 ppm and propane concentration from 100 ppm to 150 ppm.

The comparison with results from ordinary least squares regression (OLS), partial least squares regression (PLS) and the proposed MLR-RG is shown in Fig. 7, Fig. 8. Fig. 7(a) presents the actual concentrations (Column: Ground truth) and the determined concentrations of propane and isobutane using OLS, PLS and MLR-RG methods. Fig. 7 shows our proposed method of MLR-RG is more accurate than the method of OLS and PLS. The results shown in Fig. 8 indicate that the measurement errors of propane and isobutane using MLR-RG are within 10 ppm, and the measurement error of propane and isobutane using PLS or OLS are determined to be 40 ppm, 25 ppm. The measurement accuracy of propane and isobutane using MLR-RG is higher than that of ordinary least squares regression and partial least squares regression by 75% (=30/40×100%) and 60% (=15/25×100%).

**Fig. 9:**
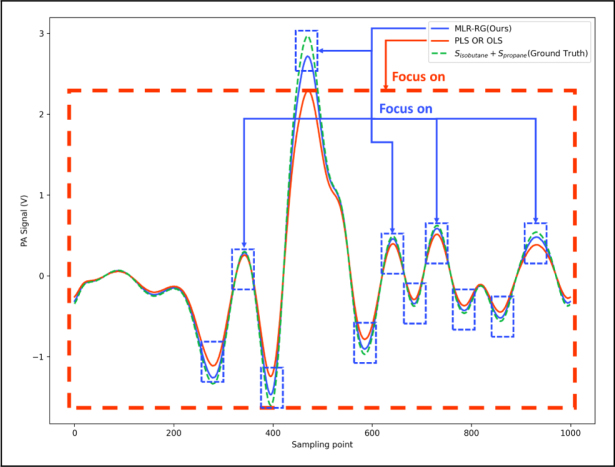
Comparison of MLR-RG and PLS, MLR-RG focus more on peak region, PLS or OLS focus on the whole region.

Least squares regression and partial least squares regression aim to solve linear correlation problems between independent and dependent variables. The calculation results using OLS and PLS are very similar as the absorption cross-section data of the three gas components are mutually independent and uncorrelated. Although partial least squares regression can suppress noise, the improvement is not effective compared to traditional least squares regression when the signal-to-noise ratio is relatively high. As shown in Fig. 9, MLR-RG tends to focus more on the second harmonic peak value, discarding data outside the peak region, which can help reduce measurement errors. The regions outside the peak cannot fully reflect changes in gas concentration, and considering these regions for regression can introduce additional errors.

### 4.3 MLR-RG based on ridge regression measuring methane, propane and isobutane

**Fig. 10:**
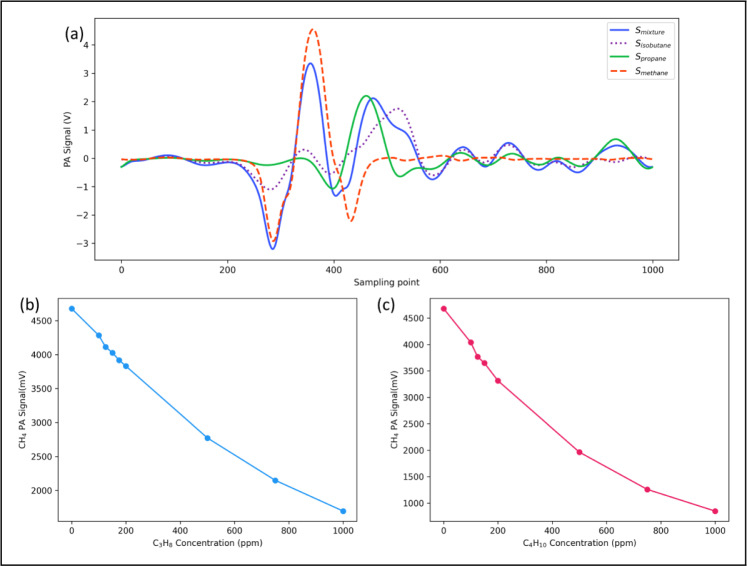
(a)Triple gas (300 ppm methane, 100 ppm propane and 150 ppm isobutane) mixing second harmonic diagram. (b)300 ppm Methane (CH_4_) PA signal of different concentration of propane (C_3_H_8_), during the measurement, the methane concentration remains unchanged, and the propane concentration is varied by changing the flow rate ratio of nitrogen and propane. (c) 300 ppm Methane (CH_4_) PA signal of different concentration of isobutane (C_4_H_10_), during the measurement, the methane concentration remains unchanged, and the isobutane concentration is varied by changing the flow rate ratio of nitrogen and isobutane.

Fig. 10(a) shows the recorded Photoacoustic signal of 300 ppm methane, 100 ppm propane, 150 ppm isobutane and their mixtures.

The concentrations determined by OLS and PLS algorithms are calculated using the mixture waveforms. Since the peak positions of the harmonics of methane and propane/isobutane are different, it is not possible to simply extract the harmonic peak data for calculations. For propane and isobutane concentrations, MLR-RG is performed on the spectra signal of propane and isobutane outside the region of the second harmonic of methane. The harmonic signals of propane and isobutane are removed from the PA signal of mixture to obtain the methane PA signal for retrieving methane concentration. For the measurement of methane, a new phenomenon was observed: the amplitude of the PA signal of methane decreased significantly when propane or isobutane is introduced.

**Fig. 11:**
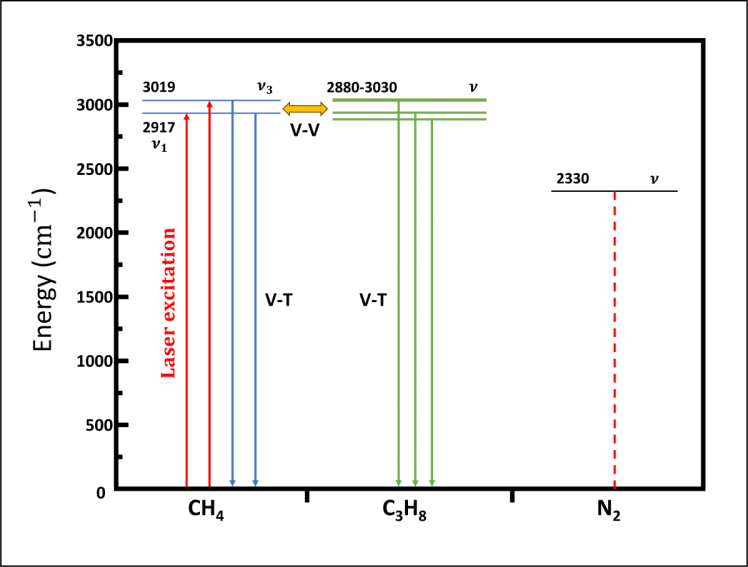
Scheme of the vibrational energy-level of CH_4_-C_3_H_8_-N_2_ system. In this process, V–T represents the vibrational-to-translational relaxation, while V–V represents the vibration-vibration transfer.

In a gas mixture of CH_4_-C_3_H_8_-N_2_, the generation rate of the photoacoustic signal of methane depends on the different relaxation times of collision substances and the V–V processes between different molecules. Fig. 11 demonstrates the vibrational energy levels of methane, propane [52], and nitrogen, while the Table 3 provides existing relaxation rate parameters.

Therefore, the relaxation time of methane can be expressed as:

$$\tau_{{\mathrm{CH}}_{4}}^{-1}=\tau_{V-T,{\mathrm{CH}}_{4}}^{-1}+\tau_{V-V,{\mathrm{CH}}_{4}}^{-1}$$

(11) $$\tau_{V-T,{\mathrm{CH}}_{4}}^{-1}=p_{N_{2}}k_{11}+p_{{\mathrm{CH}}_{4}}k_{12}+p_{C_{3}H_{8}}k_{13}$$

$$\tau_{V-V,{\mathrm{CH}}_{4}}^{-1}=p_{C_{3}H_{8}}k_{3}$$

Due to the significantly higher concentration of nitrogen (p=0.9996atm) compared to methane (p=0.0002atm) and propane (p=0.0002atm), the equation can be simplified to:

(12) $$\tau_{{\mathrm{CH}}_{4}}^{-1}\approx p_{N_{2}}k_{11}$$

The relaxation time of propane can be expressed as:

(13) $$\tau_{C_{3}H_{8}}^{-1}=p_{N_{2}}k_{21}+p_{{\mathrm{CH}}_{4}}k_{22}+p_{C_{3}H_{8}}k_{23}\approx p_{N_{2}}k_{21}$$

After calculation, the relaxation rate of methane is about $8\times 10^{4}\;s^{-1}atm^{-1}$, while the relaxation rate of propane is faster than $8\times 10^{4}\;s^{-1}atm^{-1}$. The relaxation rate of propane is faster than that of methane. Therefore, the decrease in the methane photoacoustic signal is not due to a slow relaxation rate, but rather the mutual interference between the photoacoustic signals of methane and propane.

By introducing propane and isobutane, the change of methane PA signal amplitude were observed as shown in Fig. 10(b)(c), and a linear correction was obtained as follows:

$$S_{{\mathrm{CH}}_{4}Processed}=S_{{\mathrm{CH}}_{4}}-{ɛ}_{3}\times C_{C_{3}H_{8}}$$

(14) $$S_{{\mathrm{CH}}_{4}Processed}=S_{{\mathrm{CH}}_{4}}-{ɛ}_{4}\times C_{C_{4}H_{10}}$$

**Table 3:** Relaxation rates of CH_4_, C_3_H_8_ with different collisional partners.

Reaction	Rate ($s^{-1}atm^{-1}$)	Ref.
V-T relaxation		
CH_4_ ($\nu_{1}$)+N_2_→ CH_4_+N_2_	$k_{11}=8\times 10^{4}$	[53]
CH_4_ ($\nu_{1}$)+CH_4_→ CH_4_+CH_4_	$k_{12}=8\times 10^{5}$	[53]
CH_4_ ($\nu_{1}$)+C_3_H_8_→ CH_4_+C_3_H_8_	$k_{13}=-$	–
C_3_H_8_ (ν)+N_2_→ C_3_H_8_+N_2_	$k_{21}>k_{11}$	[54], [55], [56]
C_3_H_8_ (ν)+CH_4_→ C_3_H_8_+CH_4_	$k_{22}=-$	–
C_3_H_8_ (ν)+C_3_H_8_→ C_3_H_8_+C_3_H_8_	$k_{23}=6.7\times 10^{7}$	[57]
V-V relaxation		
CH_4_ ($\nu_{1}$)+C_3_H_8_→ CH_4_+C_3_H_8_ ($\nu_{1}$)	$k_{3}=-$	–

**Fig. 12:**
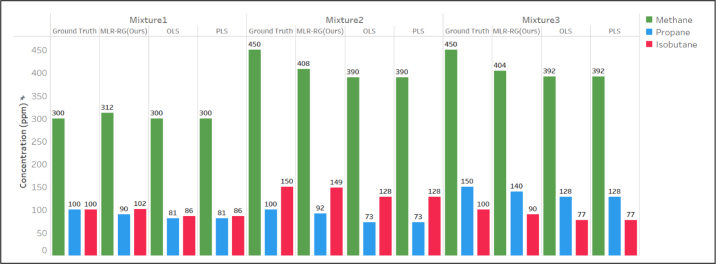
Comparison diagram of detection results of methane, propane, and isobutane three gases.

$S_{{\mathrm{CH}}_{4}}$ represents the second harmonic signal of methane in its pure state, and $S_{{\mathrm{CH}}_{4}Processed}$ represents the second harmonic signal processed through data processing and correction. $C_{C_{3}H_{8}},C_{C_{4}H_{10}}$ represent the concentrations of propane and isobutane in ppm. Through fitting, ${ɛ}_{3}$=−4.322, ${ɛ}_{4}$=−6.891 with units of mV/ppm, were obtained, which indicates that a decrement of 4.322 mV/6.891 mV in the amplitude of the second harmonic of methane is caused by 1 ppm increment of the concentration of propane/isobutane. Using this method, the calculated concentration values of methane are corrected, and the comparison results are shown in Fig. 12. The OLS and PLS algorithms have measurement errors of 30 ppm for propane, 25 ppm for isobutane, and 60 ppm for methane. The MLR-RG algorithm can reduce the measurement errors of propane and isobutane within 10 ppm and that of methane within 50 ppm compared to the two regression methods.

**Fig. 13:**
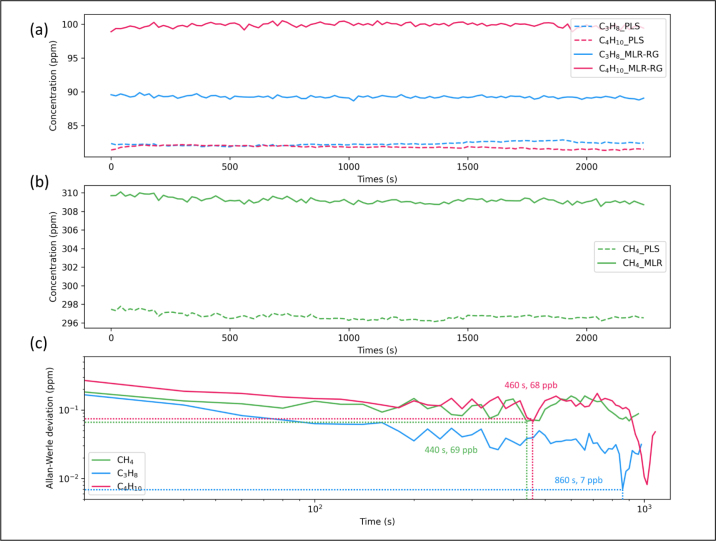
Continuous measurement results of 300 ppm methane, 100 ppm propane, 100 ppm isobutane and Allan–Werle deviation. (a)measurement results of 100 ppm propane, 100 ppm isobutane using MLR-RG and PLS. (b) measurement results of 300 ppm methane using MLR-RG and PLS. (c) Allan–Werle deviation analysis for methane, propane and isobutane using MLR-RG and plot as a function of integration time.

Continuous measurements of mixture of 300 ppm methane, 100 ppm propane, and 100 ppm isobutane (conducted every 20 s) show the concentration changes and Allan deviation in Fig. 13. MLR-RG has a limit of detection (LOD) of 440 s and 69 ppb for methane, 860 s and 7 ppb for propane, and 460 s and 68 ppb for isobutane.

## 5 Discussion and conclusion

Note that water vapor will interfere the measured concentrations of these gases. To eliminate such an interference, one can either (1) use a desiccant dryer at the gas inlet, or (2) obtain the calibration curves under different the water vapor concentrations and then measure the water vapor concentration with a humidity sensor; or (3) determine the water vapor concentration together with the concentrations of these gases by using e.g. an AI algorithm. Nevertheless, the present paper mainly focuses on the processing of broadband complex spectral signals.

A new concentration calculation method using MLR-RG is proposed. This method accurately determined the gas concentration with serious aliasing interference. Without using time-division multiplexing technology, a single DFB laser and a single resonator are combined to realize multi-gas sensing. By using the proposed algorithm based on MLR-RG. The measurement accuracy of propane, isobutane using MLR-RG is higher than that of ordinary least squares regression and partial least squares regression by 75%, and 60%, respectively. The detection limits of methane, propane, and isobutane are determined to be 828 ppb, 419 ppb, and 619 ppb (SNR = 1), respectively. The proposed algorithm based on MLR-RG provide a promising approach to process the broad overlapping absorption spectra for accurately retrieving hydrocarbon gases concentrations. The developed mid-infrared PA spectrophone has lower cost and higher precision, this feature may extend PAS technology to liquid concentration detection beyond gas sensing with narrowband absorption. Due to the much higher sound velocity in liquid media compared to gases, modulation frequency can be maintained at a lower level to reduce the resonant chamber volume. Since most substances in liquid environments exhibit broad spectrum absorption (rather than the narrowband absorption peaks of gases), similar to the broad spectrum absorption of propane and isobutane, the design of a specific photoacoustic cell incorporating a waterproof microphone holds potential for liquid composition measurement. Providing a new technical approach for precise liquid composition concentration measurement and non-invasive monitoring of blood component concentrations such as blood glucose and protein types and content measurement.

## CRediT authorship contribution statement

**Huaiyu Mei:** Writing – review & editing, Writing – original draft, Visualization, Software, Methodology, Data curation. **Gaoxuan Wang:** Writing – review & editing, Methodology, Conceptualization. **Yinghe Xu:** Investigation, Funding acquisition. **Haijie He:** Investigation, Funding acquisition. **Jun Yao:** Investigation, Funding acquisition. **Sailing He:** Supervision, Project administration, Funding acquisition, Conceptualization.

## Declaration of competing interest

The authors declare that they have no known competing financial interests or personal relationships that could have appeared to influence the work reported in this paper.

## Data Availability

Data will be made available on request.
